# Fully Complementary Interactions Between LmiRNA and mRNA of Human Genes

**DOI:** 10.3390/cimb48070665

**Published:** 2026-06-29

**Authors:** Anatoliy Ivashchenko, Anna Pyrkova, Raigul Niyazova, Saltanat Orazova

**Affiliations:** 1 Department of Biotechnology, Farabi University, Almaty 050040, Kazakhstan; raiguln@mail.ru; 2 Center for Bioinformatics and Nanomedicine, Almaty 050040, Kazakhstan; anna.pyrkova@kaznu.kz; 3 Department of Computer Sciences, Farabi University, Almaty 050040, Kazakhstan

**Keywords:** miRNA, mRNA, human gene, disease, diagnostics

## Abstract

Londin et al. discovered a novel group of miRNAs, referred to as LmiRNAs, whose properties had been studied little for unknown reasons. In this study, we examine fully complementary interactions between LmiRNAs and mRNAs of human genes. Using the MirTarget program, we identified a significant number of target genes showing unique interaction with LmiRNAs. Among the 3707 LmiRNAs, fully complementary binding sites (BSs) were found in the 5′UTR of 75 target genes, with their interactions exhibiting high free energy. Fully complementary LmiRNA binding sites were located within the CDS of 81 target genes, while only seven LmiRNAs were found to bind to the 3′UTR of target genes. The *KIFC3*, *PHF15*, *RPL15*, and *SNX11* genes were found to encode both LmiRNA-5p and LmiRNA-3p, which actively bind to their respective mRNAs. While the mRNA of most genes was targeted by only a single LmiRNA, the *BMP8B*, *FGFRL1*, and *SDC3* genes included mRNAs bound by the specific pair ID00121.5p and ID02992.5p. These results expand our understanding of LmiRNAs and support their potential as diagnostic and therapeutic agents for various diseases.

## 1. Introduction

For over three decades, miRNAs from the NCBI database have been extensively identified and utilized. However, target prediction programs have predominantly yielded false positive results, contributing to a noticeable decline in publications exploring the biological roles of miRNAs over the past few years. While Londin et al. previously identified 3707 novel miRNAs [1]—designated as LmiRNAs in this study—these molecules have surprisingly received little research attention. To address this gap, we aimed to elucidate the potential of LmiRNAs to modulate gene expression during protein synthesis. Our investigation yielded significant, biologically important findings regarding the properties of LmiRNAs and the precise quantitative characteristics of their interactions with human target genes.

## 2. Materials and Methods

For this study, the nucleotide (nt) sequences of 17,508 human genes were downloaded from the National Center for Biotechnology Information (NCBI, https://www.ncbi.nlm.nih.gov (accessed on 1 January 2022), and the mRNA isoforms with the largest nucleotide sequences were used. The nucleotide sequences of the 3707 LmiRNAs were taken from Londin et al. [1]. The LmiRNA binding sites (BSs) in mRNA were predicted using the MirTarget program [2], which predicts the following features of miRNA–mRNA interactions: (a) the initiation of miRNA binding to the mRNA from the first nucleotide of the mRNA; (b) the localization of the miRNA BSs in the 5′-untranslated region (5′UTR), coding domain sequence (CDS), and 3′-untranslated region (3′UTR) of the mRNA; (c) the schemes of all nucleotide interactions between the miRNA and mRNA; and (d) the Gibbs free energy (ΔG, kJ/mol) of the interaction between the miRNA and mRNA, as well as the ratio ΔG/ΔGm (%), determined for each site, where ΔGm is the free energy of miRNA binding with its fully complementary canonical nucleotide sequence. Only miRNAs whose nucleotides interact with mRNA via canonical (G-C and A-U) nucleotides with a given ΔG value were selected from the calculated data. The distance between the nucleotides was 1.04 nm for A-C, 1.03 nm for G-C and A-U, and 1.02 nm for G-U. The number of hydrogen bonds between the nucleotides was calculated as 3 for G-C, 2 for A-U, and 1 for A-C and G-U. The MirTarget program determines the hydrogen bonds between miRNA and mRNA according to the physicochemical characteristics of their nucleotide interactions [3,4,5,6]. MirTarget differs from other programs in that it determines miRNA BSs in mRNA in the following manner: it takes into account the interaction of miRNA with mRNA over the entire miRNA nucleotide sequence, then calculates the free energy associated with this interaction. Many other programs are based on searching for miRNA–mRNA binding sites using a 6–9 nucleotide-long region (seed), which contradicts the common sense of a fully complementary interaction with the entire miRNA. The complementarity of miRNA with mRNA only in the seed region leads to many false-positive sites. Table 1 shows a comparison of the results obtained with several programs. These results clearly demonstrate the inadequacy of these early programs, which led to a lack of reliable target gene localization predictions for many years.

Note that the nucleotides G, A, C, and U—which form the structural basis of RNA in microorganisms, plants, and animals—interact identically under equal conditions. Therefore, the physicochemical properties of the canonical nucleotide pairs given above do not require additional proof regarding the previously established physicochemical characteristics of their interactions. The reliability of translation suppression by miRNAs that are fully complementary to mRNAs was proven by A. Fire and C.C. Mello [7], who were awarded the Nobel Prize in 2006 for this research. Similarly, this year’s Nobel Prize focuses on the discovery of a vital regulatory mechanism used in cells to control gene activity. Genetic information flows from DNA to messenger RNA (mRNA) via a process called transcription, and then on to the cellular machinery for protein production.

## 3. Results

Interaction of LmiRNA with mRNA in the 5′UTR: A study of the interactions of all 3707 LmiRNAs with the mRNA from 17,508 human genes revealed that 145 LmiRNAs can interact with 163 target genes by forming fully complementary pairs of canonical nucleotides (i.e., G with C and A with U). The results of some of these calculations are presented in Table 2.

The first important property of LmiRNA is that 75 LmiRNA BSs were found in the 5′UTR of the target gene mRNAs. The free energy of the LmiRNA–mRNA interaction depends on both the GC content of the LmiRNA and mRNA BSs and the number of LmiRNA nucleotides. A ΔG value greater than −140 kJ/mole was detected for the interaction of 15 LmiRNAs with target gene mRNAs. The inclusion of LmiRNA BSs in the 5′UTR allows for translational termination early in the process, significantly reducing the energy expenditure associated with abortive protein synthesis. The mRNAs of the target genes *ANKRD9*, *CERK*, and *HYI* each bound two ID00121.5p binding sites in the 5′UTR region (Table 3).

Interaction of LmiRNA with mRNA in the CDS: A total of 81 genes were found to be LmiRNA targets with binding sites located in the CDS (Table 3). The free energy of the interaction between LmiRNA and target gene mRNA was greater than −140 kJ/mol, thus indicating a strong interaction, for 12 target genes. Seven target genes contained binding sites in the 3′UTR (Table 4); for these target genes, the free energy of the LmiRNA–mRNA interaction ranged from −110 kJ/mol to −132 kJ/mol, which was lower than that in the 5′UTR and CDS.

**Figure 1:**
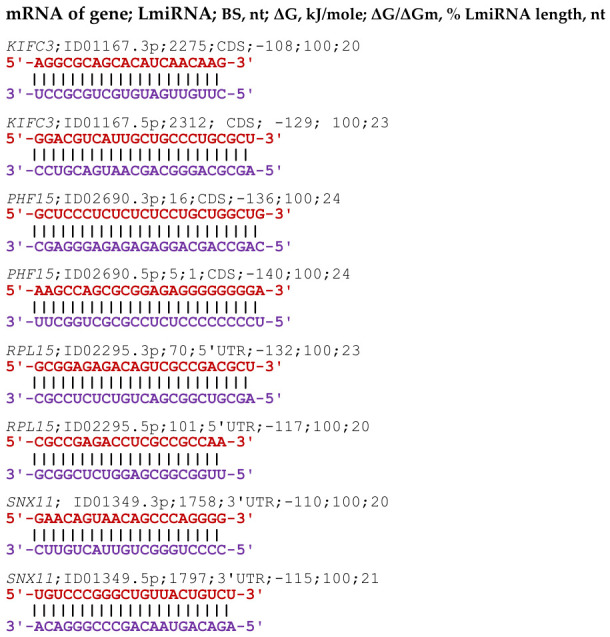
Schemes of interactions between mRNAs of the *KIFC3*, *PHF15*, *RPL15*, and *SNX11* genes and LmiRNAs encoded by these genes. Note: The mRNA nucleotides are highlighted in red. The LmiRNA nucleotides that form canonical pairs with mRNA are highlighted in violet.

Features of some target genes: We identified four genes containing regions that encode both LmiR-5p and LmiR-3p, which subsequently bind to the mRNA transcripts of their respective host genes (Figure 1). A similar architecture has been previously reported for the *RTL1* gene, which encodes four miRNA-5p and miRNA-3p pairs that target its own mRNA [8]. The evolutionary or physiological purpose for a gene encoding an LmiRNA that explicitly suppresses its own expression remains to be elucidated.

**Figure 2:**
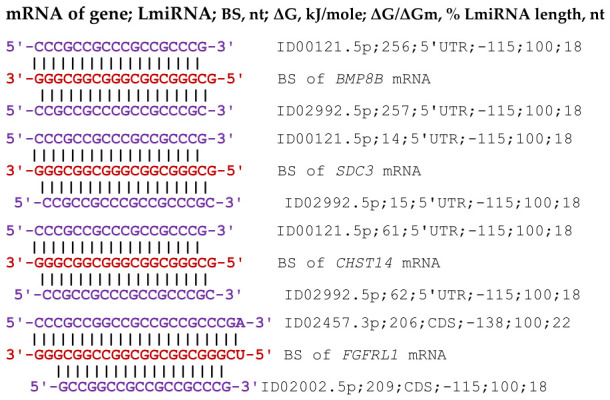
Schemes of interactions involving two LmiRNAs and one mRNA. Note: The mRNA nucleotides are highlighted in red. The LmiRNA nucleotides that form canonical pairs with mRNA are highlighted in violet.

When predicting LmiRNA-based suppression of gene expression, it is possible to detect multiple genes suppressed by a single LmiRNA. Such an example is shown in Figure 1. A given LmiRNA can suppress gene expression either alone or in combination with other LmiRNAs (Figure 2). In this article, we do not consider the functions of the genes targeted by the LmiRNAs, as this would require analyzing a large number of publications describing the involvement of genes in various diseases, many of which only establish a correlation between gene expression and disease without establishing the objective involvement of the gene in the development of a given disease.

The mRNA of the *HYI* gene was found to bind ID02992.5p at two sites. ID00121.5p and ID02992.5p were identified to bind to the miRNAs of three genes in a region of mRNA with a one-nucleotide difference in the onset of the translational sequence (Figure 2). This interaction involving two LmiRNAs may be misleading, in that it may not be possible to develop a disease prediction method by simply suppressing gene expression with only one of the LmiRNAs.

## 4. Discussion

The above results reflect only the quantitative characteristics of LmiRNA interactions with target genes, and further characterization of the involvement of target genes and the corresponding LmiRNAs that suppress their expression will require specialized, labor-intensive, and expensive studies. A gene’s involvement in a disease must be unambiguously determined, following which its expression may be suppressed or increased by increasing or decreasing the influence of certain LmiRNAs. In particular, regulation of LmiRNA expression is possible through the use of so-called miRNA-binding sponges [9].

LmiRNAs were found to interact with 163 target genes in this study, which serves as the basis for studying their involvement in the development of various diseases. For example, five genes are transcription factors (Table 2). Increased expression of *ZNF488* promotes pancreatic cancer cell proliferation and tumor development [10], and LmiRNA ID00356.3p can be used to suppress *ZNF488* gene expression. The *ZNF628* gene can serve as a marker of follicular atresia, and its expression may be regulated using ID01775.3p [11]. *ZNF750* expression may serve as a reliable prognostic biomarker for metastatic prostate cancer, which lays the foundation for the development of new biological therapies [12]. When *ZNF804B* is overexpressed on chromosome 7, it may serve as a tumor molecular marker [13]. Therefore, the obtained data on the effects of LmiRNAs on *ZNF* family transcription factors reflects the great utility of the research results.

The present study demonstrated that a single LmiRNA can reliably interact with the mRNA transcripts of one or more target genes. Conversely, multiple LmiRNAs can competitively bind to the mRNA of the same target gene. Given the vast array of potential combinatorial interactions between multiple LmiRNAs and target genes, the use of computational methods will be essential to elucidate their interaction networks. Furthermore, these predictive models must account for the specific physiological conditions and spatial contexts of LmiRNA–mRNA interactions within cells, tissues, and whole organisms.

## Figures and Tables

**Table 1:**
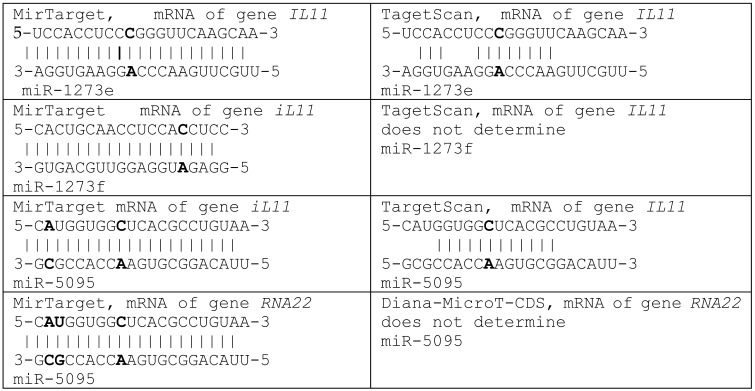
miRNA–mRNA interaction networks predicted using various software programs.

**Table 2:** Characteristics of LmiRNA interactions in the 5′UTR of mRNA in human genes.

mRNA of Gene	LmiRNA	BS, nt	ΔG, kJ/mole	mRNA of Gene	LmiRNA	BS, nt	ΔG, kJ/mole
*ADRBK2*	ID02187.5p	111	−138	*KIAA2018*	ID02368.3p	242	−140
*ALK*	ID01810.3p	52	−129	*KLHL15*	ID03422.3p	110	−123
*ANKRD9*	ID00943.3p	86	−127	*LANCL2*	ID02988.5p	425	−117
*ARSI*	ID02715.3p	511	−127	*LMX1A*	ID00230.5p	182	−129
*ASTN2*	ID03318.3p	334	−119	*LOXL4*	ID00411.5p	77	−144
*C10orf25*	ID00351.3p	70	−140	*MAP2K2*	ID01574.5p	221	−142
*C11orf87*	ID00616.5p	73	−134	*MAP3K1*	ID02634.3p	101	−129
*CAMK1D*	ID00323.3p	105	−123	*MSL1*	ID01311.3p	155	−149
*CBLL1*	ID03040.3p	184	−117	*N4BP1*	ID01157.5p	190	−125
*CD164*	ID02875.5p	68	−123	*NIPBL*	ID02626.5p	176	−123
*CDYL*	ID02781.3p	39	−125	*NLRX1*	ID00628.5p	167	−123
*CERK*	ID02992.5p	2	−115	*OGFOD3*	ID03179.5p	97	−138
*CLDND2*	D01758.3p	594	−138	*OXR1*	ID02217.5p	31	−134
*CLIC4*	ID00087.3p	34	−125	*PLA2G6*	ID02357.3p	242	−125
*CMTM6*	ID02300.3p	77	−144	*PROS1*	ID02292.5p	74	−134
*DENND6A*	ID02350.5p	11	−125	*RFTN1*	ID01075.3p	102	−140
*DUSP15*	ID02046.3p	315	−136	*RHBDL1*	ID01856.3p	243	−132
*E2F1*	ID02052.5p	85	−149	*RNF103*	ID03367.5p	37	−125
*ERCC6L2*	ID03301.3p	131	−123	*RXRA*	ID02911.5p	244	−138
*FAM131C*	ID00064.3p	1	−123	*SCAF8*	ID02686.5p	77	−121
*FAM178A*	ID00414.3p	209	−117	*SEPT8*	ID01903.3p	313	−115
*FAM81A*	ID01009.5p	14	−125	*SFT2D3*	ID00185.5p	58	−144
*GATAD2A*	ID01675.5p	109	−132	*SLC22A15*	ID01492.3p	16	−129
*GBA2*	ID03260.3p	38	−115	*SMAD2*	ID01020.5p	193	−123
*GSTZ1*	ID00910.3p	34	−123	*SMAD3*	ID02853.3p	54	−125
*HDAC4*	ID02002.5p	236	−115	*SMAP1*	ID00053.5p	265	−115
*HTRA3*	D02487.5p	37	−117	*UBIAD1*	ID00921.3p	61	−121
*ISL1*	ID02632.5p	106	−117	*UBR7*	ID03019.5p	345	−125
*KIAA1217*	ID00330.5p	145	−140				

**Table 3:** Characteristics of interactions of ID00121.5p and ID02992.5p with mRNA in the 5′UTR region of target genes.

mRNA of Gene	LmiRNA	BS, nt	ΔG, kJ/mole
*ANKRD9*	ID00121.5p	43	−115
*ANKRD9*	ID00121.5p	50	−115
*CERK*	ID00121.5p	1	−115
*CERK*	ID00121.5p	8	−115
*HYI*	ID00121.5p	126	−115
*HYI*	ID00121.5p	133	−115
*HYI*	ID02992.5p	127	−115
*HYI*	ID02992.5p	134	−115

**Table 4:** Characteristics of LmiRNA interactions with CDS and 3′UTR mRNAs of target genes.

mRNA of Gene	LmiRNA	BS, nt	ΔG, kJ/mole	mRNA of Gene	LmiRNA	BS, nt	ΔG, kJ/mole
*AATK*	ID01431.3p	2178	−142	*LHX4*	ID00245.3p	558	−123
*ADAMTS8*	ID00648.5p	821	−136	*LONRF2*	ID01873.3p	654	−132
*ADRA1B*	ID02729.5p	1320	−121	*LRRC26*	ID03389.3p	706	−121
*ANGPTL4*	ID01593.5p	259	−134	*MAP3K6*	ID00093.5p	542	−134
*APC2*	ID01540.3p	3174	−129	*METRNL*	ID01458.5p	187	−146
*APRT*	ID01212.5p	205	−110	*MIB2*	ID00017.3p	1432	−127
*ARHGEF17*	ID00592.3p	5054	−134	*MMP17*	ID00794.3p	223	−123
*ARID1B*	ID02914.3p	589	−125	*MMP24*	ID01804.3p	35	−146
*BPTF*	ID01377.3p	295	−127	*MORC4*	ID03448.3p	287	−136
*C10orf95*	ID00424.5p	845	−115	*MROH8*	ID02061.3p	188	−129
*C19orf21*	ID01521.3p	1055	−125	*MSH3*	ID02653.3p	464	−132
*C2CD4D*	ID00202.5p	1225	−146	*MXRA8*	ID00014.3p	210	−121
*C9orf66*	ID03226.3p	742	−123	*MYBBP1A*	ID01242.3p	2597	−138
*CACNA1B*	ID03398.5p	2997	−123	*MYO3A*	ID00333.3p	5184	−121
*CAMSAP1*	ID03370.5p	63	−121	*NEURL1B*	ID02740.3p	240	−119
*CCDC6*	ID00364.5p	246	−121	*PHLDA1*	ID00722.5p	282	−121
*CDHR5*	ID00474.3p	2204	−121	*PIK3IP1*	ID02201.3p	428	−119
*CEBPB*	ID02084.3p	687	−151	*PIK3R2*	ID01662.3p	1273	−142
*CELSR1*	ID02250.3p	546	−142	*PLXNC1*	ID00731.5p	582	−121
*CELSR2*	ID00178.5p	8114	−129	*PLXND1*	ID02398.3p	1058	−123
*CHADL*	ID02231.3p	1228	−115	*POU3F1*	ID00117.5p	1220	−125
*CTF1*	ID01150.3p	610	−144	*PROB1*	ID02701.5p	808	−138
*DCAF13*	ID03178.5p	317	−134	*REPIN1*	ID03072.5p	1168	−115
*DUSP28*	ID02005.5p	669	−138	*RHBDD3*	ID02191.5p	943	−121
*E2F1*	ID02051.3p	291	−153	*RNF169*	ID00594.3p	281	−134
*EPM2A*	ID02899.3p	481	−149	*SETD9*	ID02635.5p	398	−117
*F2*	ID00524.3p	532	−119	*SHISA8*	ID02233.3p	952	−132
*FAM160B2*	ID03113.3p	2279	−125	*SLC44A2*	ID01602.5p	93	−121
*FAM8A1*	ID02789.5p	399	−132	*TONSL*	ID03220.3p	2763	−127
*FBRSL1*	ID00799.3p	2503	−129	*TPM1*	ID01010.5p	333	−123
*FCRLB*	ID00226.5p	772	−136	*TRIO*	ID02611.3p	6927	−138
*FGFRL1*	ID02002.5p	209	−115	*TTC39B*	ID03248.3p	57	−123
*FGFRL1*	ID02457.3p	206	−138	*USP22*	ID01275.5p	207	−123
*GATA5*	ID02103.5p	311	−129	*ZFP36L2*	ID01824.3p	1287	−115
*GNAS*	ID02093.5p	1626	−127	*FPM1*	ID01206.3p	2213	−142
*GP1BB*	ID02171.5p	384	−129	*ZNF488*	ID00356.3p	499	−123
*GYS1*	ID01747.5p	2354	−110	*ZNF628*	ID01775.3p	3586	−125
*HCN2*	D01804.3p	112	−146	*ZNF750*	ID01456.3p	2085	−125
*HIC1*	ID01236.5p	846	−125	*C9orf62*	ID03369.3p	1261 *	−110
*IGFBP3*	ID02982.3p	435	−123	*MAPKAPK3*	ID02335.5p	2746 *	−127
*JUND*	D01663.3p	298	−125	*MLL4*	ID01699.5p	8280 *	−117
*KCNC3*	ID01755.3p	377	−127	*MLLT1*	ID01582.3p	4023 *	−123
*KDM1A*	ID00081.3p	342	−136	*SFT2D3*	ID01905.5p	1157 *	−132
*KDM3B*	ID02695.3p	242	−138	*SOX11*	ID01787.3p	3226 *	−129
				*ZNF804B*	ID01451.3p	1957 *	−132

Note. *—3′UTR.

## Data Availability

The original contributions presented in this study are included in the article. Further inquiries can be directed to the corresponding authors.
